# Mechanism Analysis of Selective Adsorption and Specific Recognition by Molecularly Imprinted Polymers of Ginsenoside Re

**DOI:** 10.3390/polym10020216

**Published:** 2018-02-22

**Authors:** Wei Zhang, Qian Li, Jingxiang Cong, Bofeng Wei, Shaoyan Wang

**Affiliations:** 1 School of Chemical Engineering, University of Science and Technology, Anshan 114051, China; askdzw@ustl.edu.cn (W.Z.); double2000li@163.com (Q.L.); congjingxiang@126.com (J.C.); askdwbf@163.com (B.W.); 2 Liaoning Provincial Key Laboratory of Fine Separation Technique, University of Science and Technology, Anshan 114051, China

**Keywords:** molecularly imprinted polymers, ginsenoside Re, thermodynamics, kinetics, mechanism

## Abstract

In this article, the molecularly imprinted polymers (MIPs) of ginsenoside Re (Re) were synthesized by suspension polymerization with Re as the template molecule, methacrylic acid (MAA) as the functional monomers, and ethyl glycol dimethacrylate (EGDMA) as the crosslinker. The MIPs were characterized by Fourier transform infrared spectroscopy (FTIR), Field emission scanning electron microscopy (FESEM), and surface porosity detector, and the selective adsorption and specific recognition of MIPs were analyzed using the theory of kinetics and thermodynamics. The experimental results showed that compared with non-imprinted polymers (NIPs), MIPs had a larger specific surface area and special pore structure and that different from the *Langmuir* model of NIPs, the static adsorption isotherm of MIPs for Re was in good agreement with the *Freundlich* model based on the two adsorption properties of MIPs. The curves of the adsorption dynamics and the lines of kinetic correlation indicate that there was a fast and selective adsorption equilibrium for Re because of the affinity of MIPs to the template rather than its analogue of ginsenoside Rg1 (Rg1). The study of thermodynamics indicate that the adsorption was controlled by enthalpy and that MIPs had higher enthalpy and entropy than NIPs, which contributed to the specific recognition of MIPs.

## 1. Introduction

Molecularly imprinted polymers (MIPs) are widely used for the specific recognition of template molecules [1,2,3,4]. Usually, MIPs are obtained by following steps. First, the monomers are arranged around the template molecules based on covalent structure or molecular self-assembly. Second, the organized architecture is achieved by the photo- or thermal polymerization in the presence of a crosslinker. Third, the binding sites, which are complementary to the template molecules in terms of size, structure, and site of the functional groups, are formed after the removal of the template by chemical reaction or extraction [5]. Therefore, the MIPs obtained have the characteristics of selectivity and recognition of the desired target [6,7,8]. Compared with some natural biomolecular recognition systems, such as antibody and antigen, receptor and ligand or enzyme and substrate, the MIPs have some significant advantages including high resistance to temperature, pressure, acid, alkali, metal ion, and organic solvents. Therefore, the MIPs can be applied not only as a substitute material for natural biological molecules but also as the separation materials under a variety of complex conditions [9,10,11].

Ginsenoside Re (CAS:51542-56-4, Re) is a tetracyclic triterpene derivative, which exists mainly in *Panax ginseng C. A. Mey* and *Panax quinquefolium L*. of the family Araliaceae [12]. It has been declared to have antioxidant effects and antihyperlipidemic efficacies [13], as well as being neuroprotective [14], strengthening immunity, and improving memory [15,16]. In recent years, the extraction and purification of ginsenoside Re from ginseng have been widely addressed in the field of separation. There are several reports on MIPs being used for the specific recognition of ginsenoside Rb1 or Rg1 from the complex extract of ginseng [17,18,19]. In order to separate Re with higher selectivity, Re was used as a template molecule in this paper.

As a widely used material, MIPs and their identification mechanism for their template have been reported in depth [20,21,22]. It is generally believed that the molecular recognition and selective adsorption are essentially the result of the complementation of the structure and function between the imprint and target molecules. The regular arrangement of functional monomers in the polymerization provides a binding space for the template, enabling the MIPs to selectively absorb imprinting molecules. The shape of the MIPs can be considered as a molecular sieve, allowing molecules that are imprinted or smaller than the template into the imprint network [23]. The adsorption mechanism of MIPs is similar to the hypothesis of the “lock and key” of an enzyme and its substrate interaction, which is a very famous principle of the adsorption mechanism of MIPs [24]. For different template molecules of MIPs, there are different adsorption models, which include the *Langmuir*, *Freundlich*, or *Langmuir-Freundlich* isotherm [25,26,27]. Although those theories provide a logical explanation for the universal application of MIPs [28,29], the conclusions are usually qualitative, modal, or theorized. In fact, there are certain limitations in some special cases such as natural low molecular weight organics and its imprinted polymers [30].

The work of this paper focused on the adsorption thermodynamics and adsorption kinetics, which help to understand the mechanism of selective adsorption and specific recognition by MIPs. The imprinted polymers were prepared and used as a specific adsorbent for ginsenoside Re using methacrylic acid (MAA) as the monomers. For a contrastive study, the ginsenoside Rg1 (CAS: 22427-39-0, Rg1), an analogue of Re, was selected. The mechanisms of selective adsorption and specific recognition of MIPs were evaluated by material characterization and adsorption experiments. The MIPs exhibited significant adsorption and recognition performances compared to the NIPs. The purpose of this article is to provide a support for further understanding the mechanism of MIPs.

## 2. Experimental

### 2.1. Instruments and Reagents

The functional groups of polymers were characterized by Fourier transform infrared (FTIR) (Nicolet IS10, Thermo Fisher, Waltham, MA, USA). Field emission scanning electron microscopy (FESEM, operated at 2.0 kV, Oberkochen, Germany) was used to characterize the morphologies and structures of the polymers. All of the chromatographic analysis data of Re and Rg1 were obtained using an LC-10AT system (Shimadzu, Kyoto, Japan). In addition, the physical adsorption parameters of the polymers were obtained using the V-sorb 2800P analyzer (Gold APP Co., Beijing, China). The UV curing machine (VIPUV Co., Guangzhou, China) was used for photo-polymerization.

**Figure 1:**
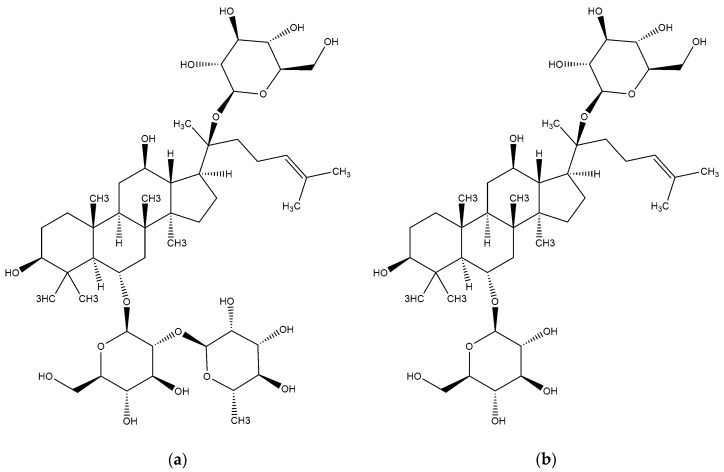
Structures of ginsenoside Re (**a**) and ginsenoside Rg1 (**b**).

Ginsenoside Re and ginsenoside Rg1 (purity > 98%) were purchased from Chengdu Must Bio-Tech. Co., Ltd. (Chengdu, China). Crude extract of ginsenoside was obtained from Zixin SGT Bio-Tech. Co., Ltd. (Tongliao, China). Structures of Re and Rg1 are shown in Figure 1. Methacrylic acid (MAA), ethyl glycol dimethacrylate (EGDMA), and poly (vinyl alcohol)-1788 (PVA-1788) were purchased from Sinopharm Chem. Reagent Co., Ltd. (Shanghai, China). EGDMA was washed with a solution of 10% NaOH, saturated solution of NaCl, and distilled water, respectively. In addition, EGDMA was dried with anhydrous Na_2_SO_4_. 2,2′-azobisisobutyronitrile (AIBN) that was obtained from Sinopharm Chemical Reagent Co., Ltd. (Shanghai, China) and was recrystallized with ethanol. All other chemicals were analytical reagents and purchased from Beijing Chem. Works (Beijing, China).

### 2.2. Preparation of MIPs

**Figure 2:**
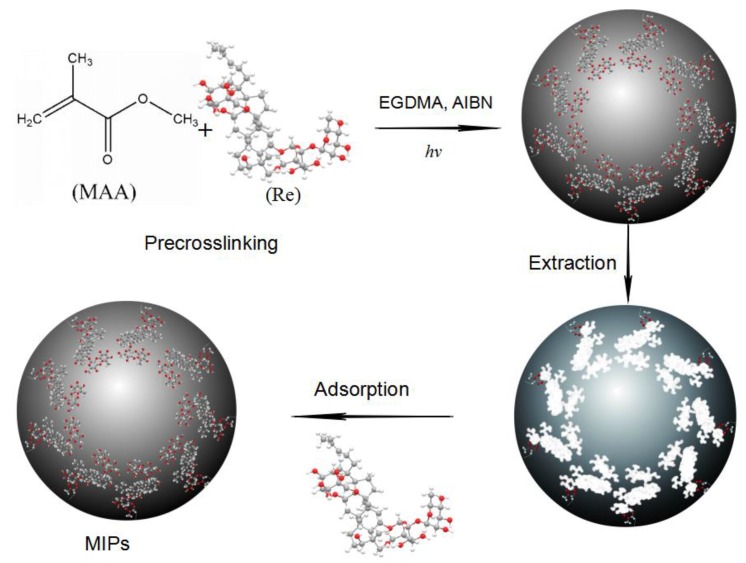
Scheme for the preparation of molecularly imprinted polymers of Re (molecularly imprinted polymers (MIPs)).

Uniformly sized polymer microspheres were synthesized by the method of suspension polymerization according to reference [31], and the MIPs were prepared by light-initiating polymerization with Re as the template, MAA as the functional monomer, EGDMA as the crosslinker, and PVA-1788 as the dispersant. Figure 2 presents the synthetic scheme of MIPs.

The imprinting process of MIP consisted of a series of complicated processes. First, 0.2 g PVA-1788 was dissolved in 30 mL water in a three-neck flask in a water bath at 80 °C. Then, the solution was cooled naturally to room temperature. Second, Re (0.90 g, 0.95 mmol) was dissolved in 10 mL chloroform and 0.450 g (5.23 mmol) MAA was added to the flask to pre-crosslink Re. Third, 2.5 g (12.5 mmol) EGDMA, 40 mg AIBN, and 50 mL toluene were added to the flask. Then, the mixed solution was stirred (600 rpm) for 24 h under constant nitrogen protection and the flask was irradiated under ultraviolet light (λ = 365 nm). Afterwards, in order to eliminate Re, the polymer was washed with a mixed solvent of methanol-acetic acid (*v/v* = 9:1) and then the residual acetic acid was removed with ethanol. Hot water (80 °C) was used last to remove the residual PVA-1788. These elimination steps for the template, acetic acid, and PVA were completed in a Soxhlet extractor, sequentially, and the time of each step was 1 h.

For comparison, non-imprinted polymer microspheres (NIPs) were also obtained by the same method except for the addition of Re.

### 2.3. Adsorption Experiments

The experiments for the adsorption kinetics were implemented at three temperatures (293, 303, and 313 K) for 5 to 60 min. The thermodynamic parameters were obtained based on the effect of these temperatures. In the experiments of the adsorption isotherm, polymers (50 mg) were added in 30% ethanol solution of Re at different concentrations (100–500 mg/L) and the mixture was shaken for 30 min. Each adsorption experiment was repeated three times and the results were obtained by HPLC, then the average was used for data analysis. The adsorption capacities of MIPs and NIPs were derived using Equation (1):

(1) $$Q_{e}=\frac{\left(C_{0}-C_{e}\right)\times V}{M}$$

where *Q_e_* (mg/g) is the capacity of the equilibrium adsorption of Re or Rg1; *C*_0_ and *C_e_* are the initial and equilibrium concentrations (mg/L) of Re; *V* (L) is the volume of the solution; and *M* (g) is the weight of the adsorbents.

In the field of molecular imprinting, for the quantitative analysis of the combination of the template and the adsorbent, the Scatchard model is used [7]:

(2) QeCe=(Qmax−Qe)KD

where *K_D_* is the dissociation constant; *Q*_max_ is the maximum adsorption capacity of Re; and *C_e_* is the equilibrium concentration of Re.

As a comparative study, the adsorption of the NIPs, together with Rg1, were carried out under the same conditions.

Analyses of Re and Rg1 were carried out using an HPLC system (LC-10AT system) with a Zorbax-C18 column (250 mm × 4.6 mm, 5 μm) and the detection wavelength was 203 nm. The mobile phase was a solution of acetonitrile–water–acetic acid (20:80:0.05, *v*/*v*/*v*) and the flow rate was 1.0 mL/L. The calibration graphs and correlation coefficients were obtained based on the quantitative determination of Re and Rg1, which were *y* = 2331.9*x* + 4.9801 (20–600 mg/L, *R*^2^ = 0.9990) and *y* = 2574.6*x* + 3.4574 (20–600 mg/L, *R*^2^ = 0.9985), respectively.

### 2.4. Adsorption Kinetics

The adsorption process was evaluated and described by adsorption kinetics [32,33]. In order to evaluate the adsorption process of Re and Rg1 on MIPs and NIPs, respectively, the first order adsorption kinetics can be preliminary used:

(3) $$\ln{\left(1-\frac{Q_{t}}{Q_{m}}\right)}_{T}=A_{r}\times \ln{\left(1-\frac{Q_{t}}{Q_{m}}\right)}_{A}$$

where Re and Rg1 are represented by the subscripts ‘*T*’ and ‘*A*’, respectively, *A_r_* is the relative adsorption rate of Re and Rg1, and *Q_t_* and *Q_m_* are the actual and the maximal amount of adsorption. The relative adsorption relationship of Re and Rg1 on MIPs and NIPs can be obtained based on Equation (3).

### 2.5. Adsorption Isotherms

Adsorption isotherms are extremely important for describing the interaction between solute and adsorbent, which indicates the distribution relationship of adsorbate between solution and adsorbent in an equilibrium state [34]. In order to investigate the adsorption capacity of an adsorbent in solution, the *Langmuir* and *Freundlich* isotherms are commonly used [35,36].

The *Langmuir* isotherm [35] is commonly used to describe the surface adsorption of single molecular layers within the adsorbent:

(4) $$\frac{C_{e}}{Q_{e}}=\frac{1}{bQ_{m}}+\frac{C_{e}}{Q_{m}}$$

where *Q_m_* (mg/g) and *Q_e_* (mg/g) are the maximum and equilibrium adsorption capacity of the adsorbent, respectively; *b* (L/mg) is the adsorption equilibrium constant; and *C_e_* (mg/L) is the equilibrium concentration of Re.

However, the *Freundlich* isotherm is highly suitable for a heterogeneous surface. The equation [26] can be expressed as follows:

(5) $$\ln Q_{e}=\ln K_{d}+\frac{1}{n}\ln C_{e}$$

where *K_f_* (mg/g) and *n* are the *Freundlich* empirical parameters.

### 2.6. Thermodynamic Analysis

As generally known, the enthalpy of adsorption is a thermal effect of the adsorption process, which is usually associated with the specific adsorption of the adsorbent and adsorbate and internal energy [37]. The adsorption of MIPs is essentially the result of the induction of the template to the polymer. Therefore, the change of adsorption enthalpy can indirectly describe the inducement between MIPs and the substrate. A larger adsorption enthalpy shows a stronger binding capacity of the MIPs to the substrate. However, the adsorption entropy is a function of the disordered state before and after adsorption, which reflects the change of adsorbate from the solution to the surface of the MIPs. Consequently, according to the change of adsorption enthalpy and adsorption entropy, the adsorption process can be learned. Based on the thermodynamic theory [38], the correlation relationship of MIPs and NIPs are expressed as follows [5]:

(6) $$\ln\left(1-\frac{Q_{T}\times w}{n_{0}}\right)=\frac{\Delta H_{ad}}{RT}-\frac{\Delta S_{ad}}{R}$$

where *n*_0_ is the initial molar number of the adsorbate; *w* is the mass of MIPs; Δ*H_ad_* and Δ*S_ad_* are the adsorption enthalpy and adsorption entropy, respectively; and *T*(K) is the absolute temperature of the adsorption process.

### 2.7. Solid-Phase Extraction

A 1-mL polypropylene SPE cartridge was filled with MIPs. The imprinted SPE cartridge was successively washed with 5 mL methanol and equilibrated using 1 mL loading solvent (methanol-water = 3:7, *v*/*v*). After the crude extract solution of ginsenoside was loaded into the SPE cartridge at a flow rate of 0.2 mL/min, the moving phase of 3 mL methanol-water (*v*/*v* = 3:7) and 3 mL methanol-water-acetic acid (*v*/*v*/*v* = 5:4.5:0.5) were used to elute the impurities and Re at 0.5 mL/min, respectively. The collected elution of Re was completely volatilized under nitrogen and redissolved in methanol for analysis by HPLC.

## 3. Results and Discussion

### 3.1. Characterization of MIPs and NIPs

**Figure 3:**
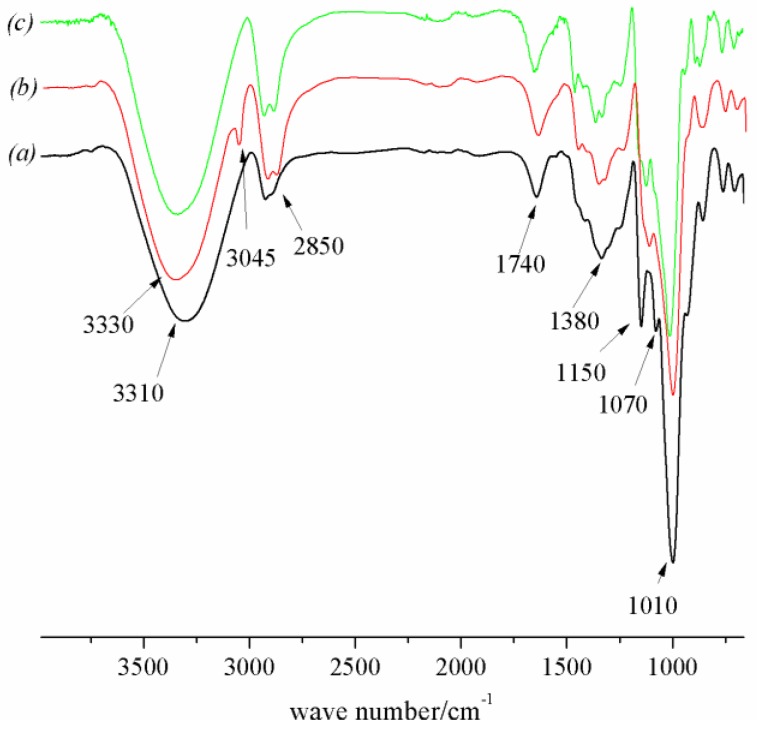
FTIR spectra of MIPs (a), MIP precursor (b), and non-imprinted polymers (NIPs) (c).

Figure 3 presents the infrared spectra of the MIPs, NIPs, and MIP precursors. The fact that the typical feature of –C=C– around 3045 cm^−1^ in Figure 3b disappeared confirmed that the MIPs (Figure 3a) and NIPs (Figure 3b) had been prepared. The stretching adsorptions at around 3310, 2850, 1740, 1380, 1150, 1070, and 1010 cm^−1^ represented the functional groups of –OH, –CH_2_–, –COO–, –CO–, –CH_3_, –COOC, –CO (*v*), and –CH (σ), respectively. For MIPs, a red shift of –OH stretching bond appeared, which related to the limitation of the template molecules during polymerization. After washing, the spectrum of MIPs became comparable to that of NIPs, indicating that almost all of the template was removed and ready for Re adsorption. Therefore, due to the binding sites being complementary to Re in terms of size and structure, MIPs had a characteristic selectivity and recognition for Re.

**Figure 4:**
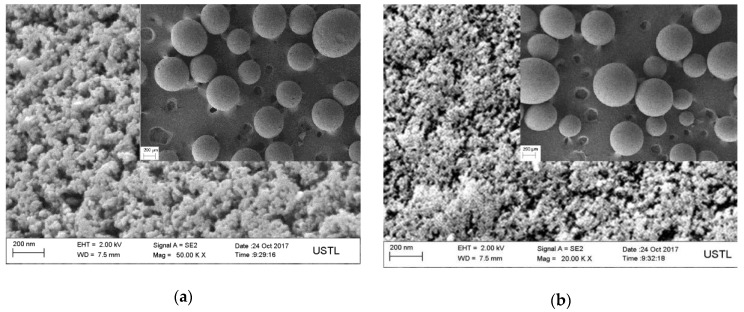
FESEM images of MIPs (**a**) and NIPs (**b**).

**Figure 5:**
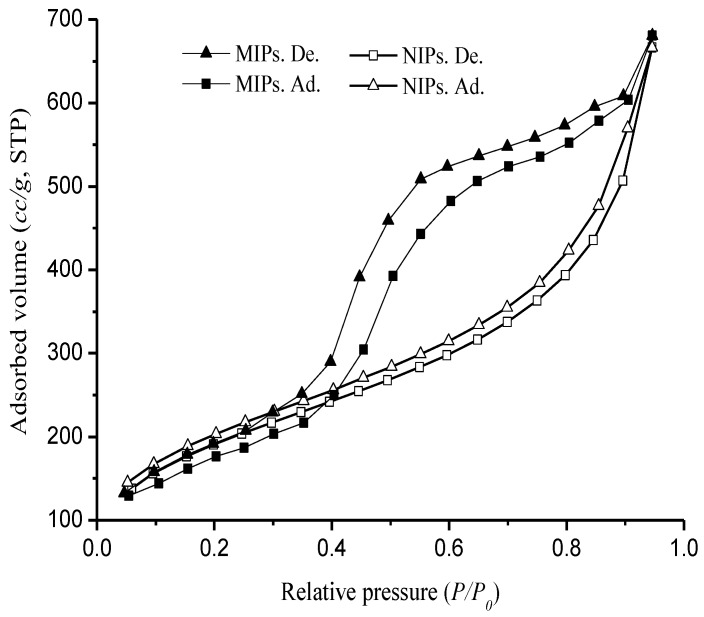
Nitrogen adsorption/desorption isotherms for MIPs and NIPs.

After polymerization, Re was eliminated after a post-processing step and micropores, as imprinted sites, were left in the MIPs. Figure 4 obviously shows that the pores were formed in the structures of MIPs and the surface of NIPs was relatively smooth. Therefore, MIPs often have a larger surface area and a rougher structure with larger cavities than those of NIPs, which is mainly due to the presence of the imprinted sites of the template molecule. Nitrogen adsorption-desorption plots of MIPs and NIPs are shown in Figure 5. The adsorption curve of MIPs did not coincide with the desorption curve and an adsorption lag was produced. The shape of the hysteresis loop reflects the pore structure in the MIPs. Curves of MIPs had a hysteresis effect [38] (type II) within the range of 0.4–0.6 (relative pressure). The hysteresis effects indicate that the uniform size and shape rule of mesoporous pores existed in the pore structure of the MIPs compared with the NIPs. In addition, the specific pore volumes, pore diameters, and surface areas of MIPs and NIPs are shown in Table 1. The results show that the specific surface area and the average pore diameter of MIPs were greater than that of NIPs. This indicates that the templates confined the shrinkage of the mesoporous pores effectively in the process of the polymerization and the synthesized MIPs had a more regular pore structure. These differences provided a complementary spatial structure for the selective recognition of the template and MIPs, and underline the indistinct impact of the template molecule on the molecularly imprint polymer.

### 3.2. The Recovery of Re

In the preparation of MIPs, that is, the polymerization of functional monomers and crosslinker, the double bonds of the reactants were broken to form the polymer matrix. Although Re has double bonds, with its large size and steric hindrance, it does not co-polymerize into a polymer. This result was proven by the above FTIR spectra of MIPs in Figure 3 where the characteristic peaks of ginsenoside Re (2950, 1650, and 1460 cm^−1^) were not found. Therefore, Re was easily washed out from the polymer matrix with a high Re recovery of 93.3%.

### 3.3. Adsorption Studies of MIPs and NIPs

**Figure 6:**
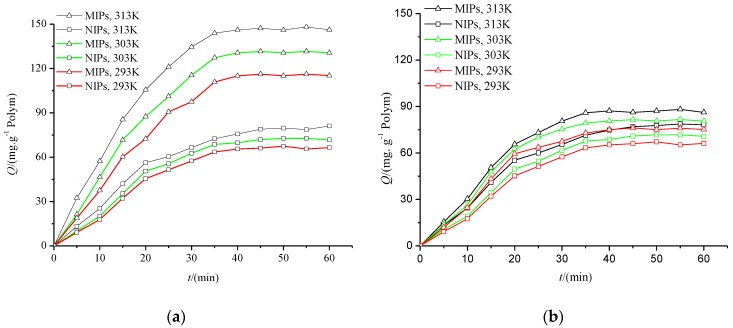
The adsorption dynamics curves of the MIPs and NIPs ((**a**), Re; (**b**), Rg1). Adsorption conditions: 20 mL of 0.4 mg/mL solutions (methanol) of Re or Rg1 with 50 mg of MIPs or NIPs.

The adsorption kinetic curves (Figure 6) for the adsorption of Re and Rg1 on MIPs and NIPs show that the amount of adsorption increased with an increase in temperature or adsorption time. There was a narrow difference between the Re and Rg1 with NIPs as the adsorbent. However, using MIPs, the adsorption capacity of Re was much larger than NIPs and larger than that of Rg1. The result indicates the selective adsorption for the template by the MIPs.

**Figure 7:**
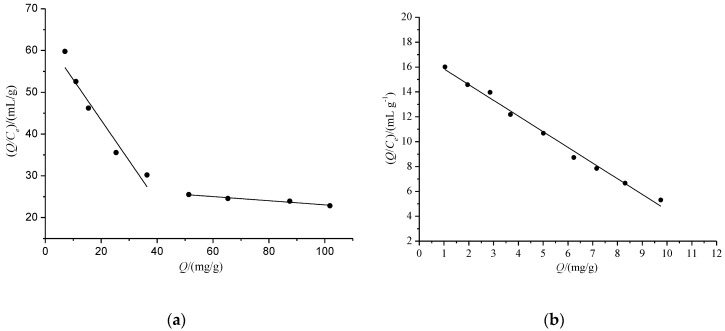
Scatchard plots of MIPs (**a**) and NIPs (**b**) for Re.

Figure 7 is the Scatchard curves of Re on MIPs and NIPs. It shows that there were two discontinuous lines of MIPs, which indicate there were two adsorption properties on MIPs, including the higher adsorption combining sites with the fitting equation of *Q*/*C_e_* = −0.972*Q* + 62.77 (*R*^2^ = 0.8852) and the lower adsorption combining sites with the fitting equation of *Q*/*C_e_* = −0.049*Q* + 27.93 (*R*^2^ = 0.9527). According to the two regression lines, *K_D_* and *Q*_max_ were 1.0288 mg/mL and 64.58 mg/g for first receptor sites and 20.41 mg/mL and 570.05 mg/g for the second receptor sites, respectively. On the contrary, one line of NIPs indicate only one type of adsorption function of NIPs; the fitting equation was *Q*/*C_e_* = −1.260*Q* + 17.10 (*R*^2^ = 0.9906) and *K_D_* and *Q*_max_ were 0.7937 mg/mL and 13.57 mg/g, respectively. The results show that only the adsorption of MIPs had a significant positive departure for Re.

### 3.4. Kinetic Consideration

**Figure 8:**
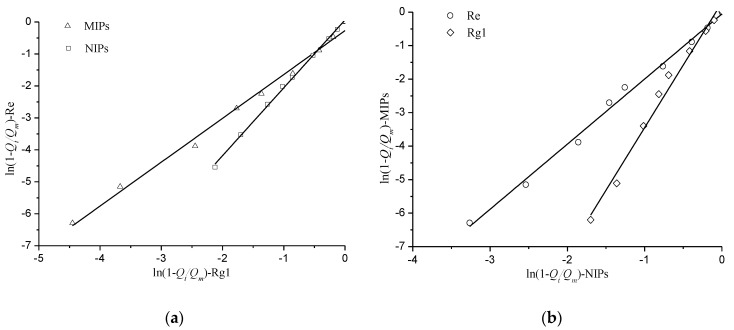
Kinetically correlating the relative adsorption of Re and Rg1 (**a**) and the relative adsorption of MIPs and NIPs (**b**).

Based on Equation (3), the plots of kinetic correlations for the relative adsorption of Re and Rg1 and the relative adsorption of MIPs and NIPs are shown in Figure 8. The relevant parameters of the linear fitting equation are shown in Table 2. The typical nonspecific adsorption of NIPs can be revealed because the *A_r_* (slope) value was approximately 1 (Figure 8a). For MIPs, however, the slope shows a distinct specific adsorption. Plotting ln(1 − *Q_t_/Q_m_*)-Re versus ln(1 − *Q_t_/Q_m_*)-Rg1 is normally expected to give a straight line. The capacity of adsorption for Re was 1.27 times that of Rg1 and the relative adsorption rate for Re was 1.28 times as much as that for Rg1. The results indicate that a faster adsorption equilibrium and selective adsorption were the results of the affinity of MIPs for the template, and the specific adsorption of MIPs was the result of a greater promotion in the process of imprinting.

### 3.5. Adsorption Isotherms

**Figure 9:**
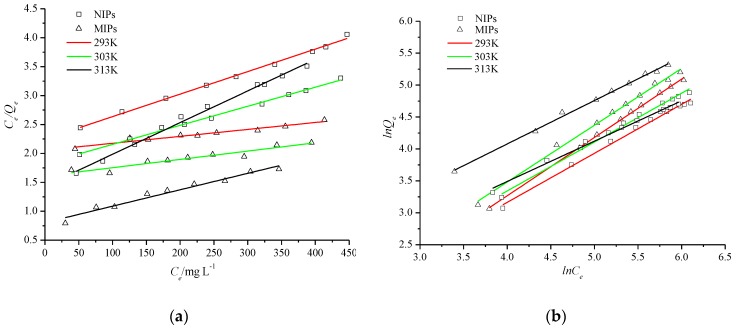
*Langmuir* (**a**) and *Freundlich* (**b**) isotherms for the adsorption of NIPs and MIPs.

Plots of *Langmuir* isotherms for the adsorption of MIPs and NIPs for Re are shown in Figure 9a, and the values of *Q_m_* and *b* were obtained from the slopes and intercepts of the plots. Similarly, plots of *Freundlich* isotherms are shown in Figure 9b and the values of *K_f_* and *n* were achieved from the intercepts and slopes of the plots. The parameters and correlation coefficients of the adsorption isotherms are tabulated in Table 3. For MIPs, the correlation coefficients (0.9949–0.9989) of the *Freundlich* model were very close to 1 and much larger than the *Langmuir* model (0.8990–0.9625), indicating that the adsorption process of the MIPs for Re was more consistent with the adsorption of a porous heterogeneous surface. However, the adsorption process of the NIPs was more suitable for the *Langmuir* model of single molecular layer adsorption. The adsorption distribution coefficient (*K_D_*) was equal to the value of *Q_e_*/*C_e_* (*C*→0). The *K_D_* values of the two cases are also listed in Table 3. The values of *K_D_* of MIPs were larger than those of NIPs and were the result of the specific recognition by MIPs for Re.

### 3.6. Thermodynamic Studies

**Figure 10:**
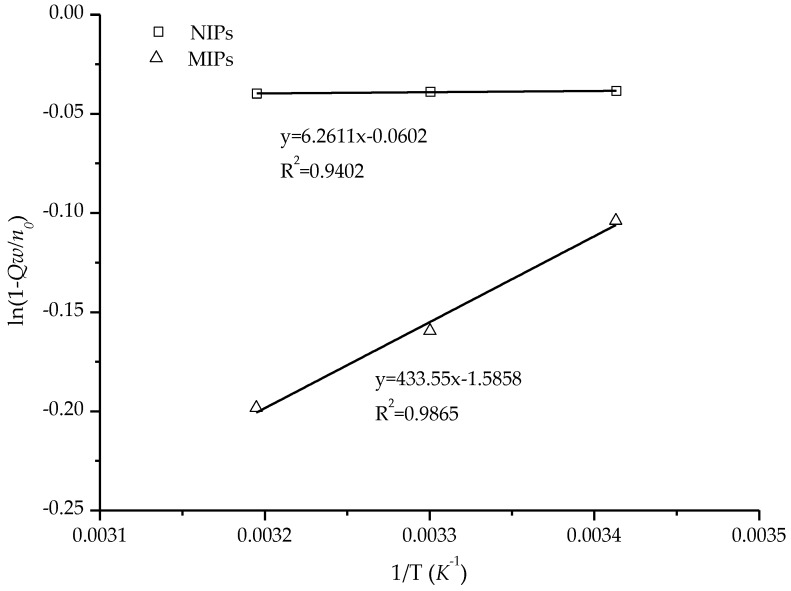
Thermodynamically correlating the relative adsorption of Re.

Based on Equation (6), the plot of ln(1 − *Q* × *w*/*n*_0_) vs. (1/*T*) is shown in Figure 10; the plots are normally expected to be a straight line. Compared with NIPs, the MIPs showed a significantly different adsorption behavior for Re. Relative to NIPs (Δ*H_ad_* = 0.0521 KJ/mol), the adsorption enthalpy (Δ*H_ad_* = 3.6045 KJ/mol) of MIPs was obviously larger. This indicates that the inducement of MIPs for Re is greater than that of NIPs. However, the adsorption entropy of MIPs (Δ*S_ad_* = 0.0132 KJ/mol·K) was less than NIPs (Δ*S_ad_* = 0.50 × 10^−3^ KJ/mol·K). The change in adsorption entropy, which reveals a difference in the adsorption behavior of Re and Rg1, may be the result of the increased interaction between the molecules due to the high specific imprint. The change in adsorption entropy can be the result of a larger restriction on molecular motions due to the adsorption. According to the results of the adsorption isotherms and kinetics, Re adsorption on MIPs was the spontaneous process of enthalpy control. Relative to the previous discussion, the obvious induction in MIPs can be considered to be the result of highly specific imprinting, which allowed the MIPs to specifically bind Re.

### 3.7. Application of MIPs-SPE for Crude Extracts

**Figure 11:**
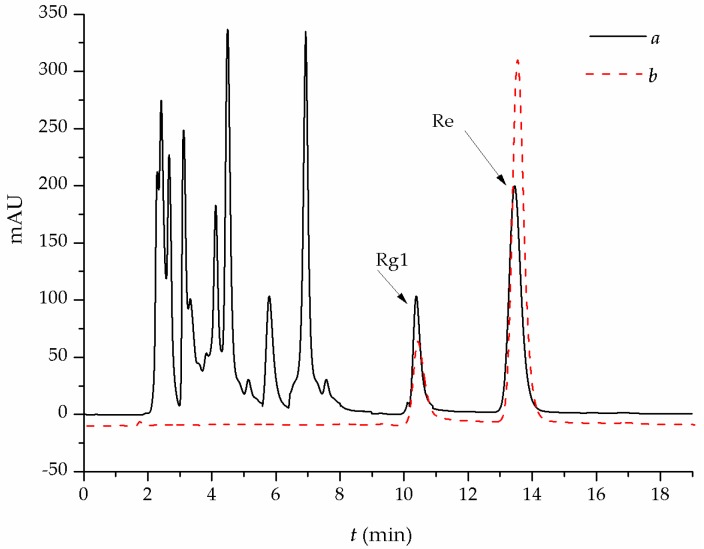
Chromatograms of (**a**) the crude extract of ginsenosides and (**b**) the collected elution with MIPs-SPE.

Figure 11a is the HPLC profile of the crude extract of ginsenoside, which shows that there were complex chromatographic peaks corresponding to Re, Rg1, and other multiple impurities. The peak area ratio of Re to Rg1 in the crude extracts was 2.47. Figure 11b is the HPLC profile of the collected elution from MIPs-SPE, which shows that there were two chromatographic peaks corresponding to Re and Rg1 and the ratio of Re to Rg1 in the collected elution was 4.21. The SPE experiment result demonstrates that the MIPs completely removed the major impurities and efficiently increased the content of Re as Re and Rg1. Although the structure difference of Re and Rg1 was only one glycoside, MIPs still exhibited good selectivity for Re. Therefore, the synthesized MIPs of Re exhibit practical value for their application to crude extracts of ginsenosides.

## 4. Conclusions

Compared with non-imprinted polymers (NIPs), the experimental results show that MIPs have a larger specific surface area and special pore structure. For ginsenoside Re, the static adsorption isotherm of MIPs was in good agreement with the *Freundlich* model based on the two adsorption properties on MIPs, and there was a fast and selective adsorption equilibrium with high enthalpy and entropy. Re adsorption on MIPs is the spontaneous process of enthalpy control. All of this information indicates that the induced molecular memory within the MIPs make the polymer selectively adsorb Re. The present investigation suggests that the molecularly imprinted polymers of ginsenoside Re can be employed as an effective adsorbent for the enrichment of Re. We believe that these results are valuable and necessary for further research on the recognition mechanism of molecular imprinting in the future.

## Figures and Tables

**Table 1:** Properties of MIPs and NIPs.

Polymer	Specific Surface Area ^a^ (m^2^·g^−1^)	Average Pore Diameter ^b^ (nm)	Specific Pore Volume ^c^ (mL·g^−1^)
MIPs	328.96	4.43	0.69
NIPs	255.77	3.78	0.62

^a^ Calculated by the Brunauer-Emmett-Teller (BET) formula; ^b^ Calculated by the Dubinin-Radushkevich (D-R) formula; ^c^ Calculated by the Barrett-Joyner-Halenda (BJH) formula.

**Table 2:** The relevant parameter of the linear fitting equation.

Object	Relevant Parameter	
	Slope	Statistics
MIPs	1.3706	0.9932
NIPs	1.0828	0.9984
Re	1.9433	0.9938
Rg1	1.5259	0.9850

**Table 3:** The isotherm parameters of MIPs and NIPs.

Polymer	*T*(K)	*Langmuir* Isotherm				*Freundlich* Isotherm			
		*Q_m_*	*b*	*K_D_*	*R*^2^	*K_f_*	*n*	*K_D_*	*R*^2^
MIPs	293	0.83	0.58	0.49	0.9483	0.68	1.10	1.07	0.9989
	303	0.71	0.87	0.62	0.8990	0.93	1.13	1.37	0.9949
	313	0.36	1.00	1.25	0.9625	1.85	1.47	1.80	0.9962
NIPs	293	0.25	1.76	0.45	0.9958	1.09	1.30	0.57	0.9941
	303	0.30	1.80	0.56	0.9951	1.34	1.32	0.77	0.9937
	313	0.39	1.83	0.70	0.9938	2.65	1.58	0.83	0.9921
